# Multilayered regulation of BDNF DNA methylation in PTSD: a review from molecular mechanisms to trans generational inheritance

**DOI:** 10.3389/fpsyt.2026.1734160

**Published:** 2026-01-22

**Authors:** Lin Wang, Bing Lv, Zihan Ma

**Affiliations:** 1 Department of Applied Psychology, School of Innovation and Entrepreneurship Education, Heilongjiang University, Harbin, China; 2 Department of Emergency, The Fourth Affiliated Hospital of Harbin Medical University, Harbin, China

**Keywords:** brain-derived neurotrophic factor (BDNF), DNA methylation, epigenetic regulation, neural plasticity, post-traumatic stress disorder (PTSD)

## Abstract

Post-traumatic stress disorder (PTSD) is a chronic psychiatric disorder triggered by a traumatic event. Its core features include intrusive flashbacks, persistent avoidance, negative cognition and mood changes, and heightened arousal. The global lifetime prevalence is approximately 3.9%, exceeding 5.0% in high-income countries and high-trauma-exposed populations. With rising incidence of natural disasters, violent conflicts, and public health incidents worldwide, PTSD has become a serious public health issue threatening people’s mental health. However, its pathogenesis remains largely unknown, specific clinical diagnostic biomarkers are lacking, and treatment efficacy varies significantly across individuals. Molecular understanding of its pathophysiology is urgently needed. Brain-derived neurotrophic factor (BDNF), a key neurotrophic factor in the central nervous system, is crucial for regulating neuronal survival, differentiation, and synaptic plasticity. Abnormal synaptic plasticity is closely associated with abnormal fear memory storage and emotional regulation impairments in PTSD patients. DNA methylation, a classic epigenetic regulatory mechanism, can inhibit transcriptional activity by modifying CpG sites in gene promoter regions. Its role in regulating BDNF gene expression has been widely demonstrated. In recent years, more epidemiological and animal studies suggest that BDNF DNA methylation may serve as a key molecular bridge between trauma exposure and the onset of PTSD. Abnormally elevated BDNF promoter methylation levels have been detected in the peripheral blood and in core brain regions(hippocampu,samygdala) of PTSD patients. Furthermore, these methylation levels can predict the risk of developing PTSD after trauma and are significantly correlated with clinical features such as impaired cortisol secretion and generalized fear memory. This study conducted a literature review, with data collected from authoritative Chinese and English databases. Chinese literature was retrieved from CNKI (China National Knowledge Infrastructure) and Wan fang Data; English literature was sourced from PubMed and Web of Science. The search was restricted to articles published prior to December 2025, focusing on case-control studies investigating the association between BDNF DNA methylation and post-traumatic stress disorder (PTSD). This review followed a structured, but not systematic, search strategy. We focus on the specific molecular pathways by which BDNF DNA methylation contributes to PTSD pathogenesis by influencing neural circuit plasticity, hippocampal function, and hypothalamic-pituitary-adrenal (HPA) axis homeostasis. We also summarize its potential for application in the development of diagnostic biomarkers and targeted interventions for PTSD. We also outline cutting-edge research directions driven by emerging technologies such as single-cell sequencing and epigenetic editing. This article aims to provide theoretical references for a deeper understanding of the pathogenesis of PTSD and promote clinical translational research.

## 1 Introduction

Posttraumatic stress disorder (PTSD) is a psychiatric disorder triggered by severe traumatic events. Its prevalence in the general population is 6% to 8%, but in groups that have experienced severe psychological trauma, such as veterans, refugees, and victims of attacks, the rate may increase to 25%. Its core features include abnormal consolidation, extinction disorder, and generalization of pathological fear memories, and clinical manifestations include repeated flashbacks of traumatic memories, hypervigilance, and emotional dysregulation (1). In China, the first national mental health survey (sample size n = 32,552) recorded a lifetime prevalence of 0.4% and a 12-month prevalence of 0.2% for posttraumatic stress disorder (PTSD), but these data may underestimate the true burden due to underreporting and diagnostic differences (2). In addition to psychological impact, PTSD is also associated with comorbidities such as anxiety, depression, type 2 diabetes, and cardiovascular disease, which further impair patients’ quality of life and cause significant social costs (3).

Researchers have found that BDNF gene methylation may be closely related to the occurrence and development of PTSD. Brain-derived neurotrophic factor (BDNF) is an important neuron-specific transcription factor that plays an important role in neuronal growth, differentiation and synaptic plasticity (4). BDNF gene expression is regulated by its DNA methylation, which is a common epigenetic modification that affects gene transcription and expression by adding methyl groups to DNA bases. The DNA methylation status of the BDNF gene can regulate its transcriptional activity, thereby affecting the production of BDNF protein. Changes in BDNF expression and signal transduction can affect neural-related cognitive dysfunction.

Despite growing evidence implicating BDNF methylation in PTSD pathophysiology, three key gaps remain. First, prior studies have presented conflicting results—some reporting hypermethylation, others hypomethylation—potentially reflecting differences in cell type composition, genomic loci examined, medication status, and developmental timing of trauma exposure. Second, the causal pathways by which BDNF methylation translates into the multisystem (molecular, cellular, circuit, endocrine) phenotypes of PTSD remain incomplete. Third, the therapeutic value of targeting BDNF methylation—either pharmacologically or through epigenome editing—has yet to be systematically evaluated in preclinical or clinical settings.

Here, we integrate longitudinal cohorts, translational neuroimaging, single-cell epigenomics and germline transmission studies to provide a unifying framework for BDNF DNA methylation in PTSD. We further outline how BDNF methylation interacts with glucocorticoid signaling to create a self-reinforcing cycle of impaired synaptic plasticity and HPA axis dysregulation. Finally, we evaluate emerging intervention strategies—from DNMT and HDAC inhibitors to CRISPR-dCas9-based epigenetic editing—and discuss their potential for precision medicine and prevention of intergenerational trauma.

## 2 Thematic sections

This study conducted a literature review, with data collected from authoritative Chinese and English databases. The Boolean search strategies adopted for literature collection were as follows:(1) Chinese: (“脑源性神经营养因子” OR “BDNF”) AND (“DNA 甲基化” OR “甲基化”) AND (“PTSD” OR “创伤后应激障碍”);(2) English: (“Stress Disorders, Post-Traumatic” OR “Post-Traumatic Stress Disorder” OR “Stress Disorder, Post-Traumatic” OR “Post Traumatic Stress Disorder” OR “Neuroses, Post-Traumatic” OR “Neuroses, Post Traumatic” OR “Post-Traumatic Neuroses” OR “PTSD” OR “Stress Disorder, Post Traumatic” OR “Post-Traumatic Stress Disorders” OR “Post Traumatic Stress Disorders” OR “Posttraumatic Stress Disorders” OR “Posttraumatic Stress Disorder” OR “Stress Disorder, Posttraumatic” OR “Stress Disorders, Posttraumatic” OR “Neuroses, Posttraumatic” OR “Posttraumatic Neuroses” OR “Acute Post-Traumatic Stress Disorder” OR “Acute Post Traumatic Stress Disorder” OR “Chronic Post-Traumatic Stress Disorder” OR “Chronic Post Traumatic Stress Disorder” OR “Delayed Onset Post-Traumatic Stress Disorder” OR “Delayed Onset Post Traumatic Stress Disorder”) AND (“DNA Methylation” OR “DNA Methylations” OR “Methylation, DNA” OR “Methylations, DNA” OR “Methylations” OR “Methylation”) AND (“Brain-Derived Neurotrophic Factor” OR “Brain Derived Neurotrophic Factor” OR “Factor, Brain-Derived Neurotrophic” OR “Neurotrophic Factor, Brain-Derived” OR “BDNF”).Based on existing research progress, this article reviews the biological functions of BDNF and its DNA methylation regulatory mechanisms. A structured literature search was performed to ensure broad coverage, but no systematic quality appraisal or meta-analysis was conducted. The synthesis is conceptual and interpretive, focusing on mechanisms and emerging evidence. The specific details are as follows:

### 2.1 Neurobiological mechanisms of PTSD

The biological basis of PTSD development remains incompletely understood. Exploring its underlying brain mechanisms will not only fill gaps in pathophysiological research but also provide critical theoretical foundations for developing effective early intervention and prevention strategies. Functional magnetic resonance imaging (fMRI) studies have shown that technology, as an important tool for analyzing brain dysfunction, has revealed the association between PTSD and functional changes in specific brain regions in many studies. Among them, subcortical structures such as the medial prefrontal cortex, anterior cingulate cortex, amygdala and hippocampus have been confirmed to be core brain regions involved in the pathological process of PTSD (5). Zhong Y et al. (6) conducted a resting-state fMRI study on 14 PTSD outpatients and 14 age- and sex-matched healthy controls. The results showed that compared with healthy controls, PTSD patients had significantly increased local coherence in subcortical regions such as the amygdala, hippocampus, thalamus and putamen, while local coherence in cortical regions such as the medial prefrontal cortex and dorsolateral prefrontal cortex was significantly reduced. This imbalance in functional coherence of brain regions may be directly related to abnormal emotion regulation and cognitive control in PTSD patients. It is worth noting that the amygdala, as the core structure in the brain responsible for emotion processing, plays a key role in the emotional disturbances of PTSD patients due to its dysfunction. Furthermore, a recent review published in the New England Journal of Medicine further pointed out that four brain functions, namely emotion regulation and executive function, threat detection, situational processing, and fear learning, play an indispensable role in the development of PTSD psychopathology (5, 7). These functions, through their synergistic effects with the above-mentioned core brain regions, together constitute the key links in the brain mechanism of PTSD, providing a clear direction for subsequent targeted intervention research.

### 2.2 Association between BDNF DNA methylation and PTSD

**Figure 1:**
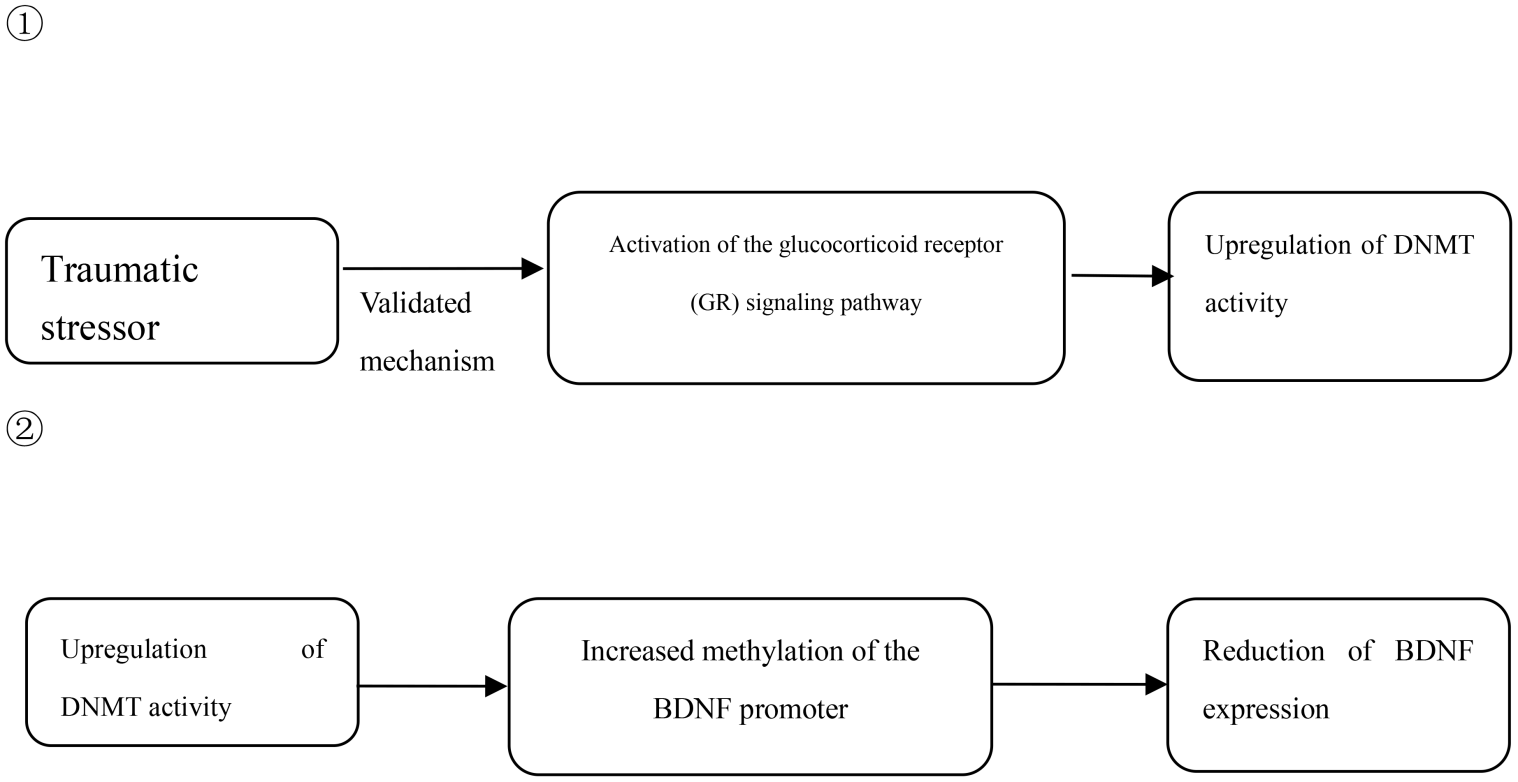
Association between BDNF DNA methylation and PTSD.

Brain-derived neurotrophic factor (BDNF) plays a key role in the pathogenesis of various neuropsychiatric disorders, including post-traumatic stress disorder (PTSD). BDNF promoter methylation, as an important epigenetic regulatory mechanism, can directly alter BDNF expression levels and its neuroprotective functions, as detailed in **Figure 1**. Numerous epidemiological studies have confirmed a close association between the methylation status of CpG sites in the BDNF gene promoter and PTSD, suggesting that methylation levels may serve as a potential biomarker for the diagnosis of PTSD. In terms of specific research evidence, Guo J et al. (8) conducted a study on 164 PTSD patients and 141 healthy controls and found that not only were the serum BDNF levels of PTSD patients significantly higher than those of the control group, but the methylation level of their BDNF promoter was also significantly higher; Lee HS et al. (9) further observed in 99 PTSD patients and 81 healthy controls that there was an interaction between traumatic experience and epigenetic methylation of BDNF, especially that childhood trauma exposure may have a long-term impact on the methylation status of BDNF; Guo JC et al. (10) conducted a study on 322 PTSD patients and 215 controls and directly showed that stressful events may be an important inducer of methylation of CpG sites in the BDNF promoter region of PTSD patients; similarly, Kim TY et al. (11) conducted a study on 126 veterans and 122 healthy controls and found that compared with those without PTSD, the methylation level of BDNF promoter in PTSD patients was significantly higher than that in PTSD patients without PTSD. Compared with subjects without combat exposure, the DNA methylation levels of four CpG sites in the BDNF promoter region of PTSD patients were significantly increased, and this high methylation state and high combat exposure were significantly correlated with the PTSD diagnosis results. However, the conclusions of the studies are not completely consistent. Voisey J et al. (12) studied 96 Vietnam veterans and found that the methylation levels of three CpG sites in the BDNF gene of PTSD patients (cg01546433, P = 0.005; cg24650785, P<0.001; cg002298481, P<0.001) were reduced, and no correlation between BDNF mRNA expression and PTSD was observed. The researchers speculated that this difference may be related to the fact that 70% of PTSD participants were taking psychotropic drugs at the time, because studies have shown that antidepressants and psychotherapy can reduce BDNF methylation levels (13). Furthermore, a longitudinal observational study further explored the relationship between DNA methylation in the BDNF gene region and the development of PTSD. The results showed that high methylation levels of BDNF DNA after a traumatic event significantly predicted the risk of PTSD. This finding provides stronger support for a possible causal relationship between methylation in the BDNF gene region and PTSD.

### 2.3 The mechanism of action of BDNF DNA methylation in PTSD

**Figure 2:**
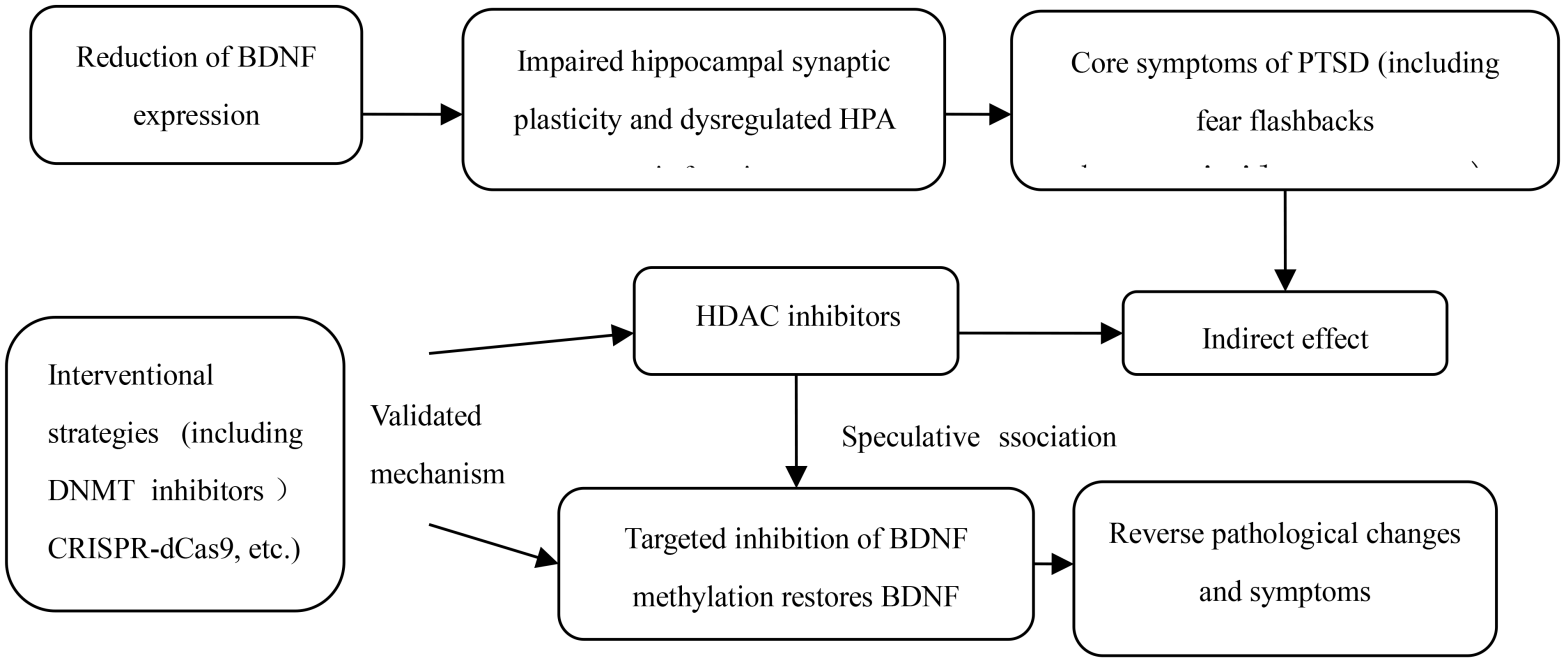
The mechanism of action of BDNF DNA methylation in PTSD.

Although epidemiological studies have confirmed a positive correlation between BDNF DNA methylation and PTSD, the underlying physiological mechanism of the association remains unclear. In animal cell models, traumatic events have been shown to significantly increase BDNF DNA methylation levels, as detailed in **Figure 2**. For example, rats exposed to early developmental stress or fear conditioning in adulthood show subtype-specific epigenetic marks, including BDNF DNA methylation. These results suggest that traumatic stress in adulthood can induce gene methylation in the central nervous system, and epigenetic changes in the BDNF gene may be one of the core molecular mechanisms of PTSD pathophysiology. Increased BDNF DNA methylation levels directly lead to decreased gene expression, which is also the most intuitive physiological mechanism of the association between the two. DNA methylation is generally associated with gene transcriptional repression, and extensive methylation can even trigger complete gene silencing. For example, the offspring of rats exposed to traumatic events prenatally have decreased BDNF expression in the amygdala and hippocampus during weaning and adulthood, which is partly mediated by increased DNA methylation of BDNF exon IV (14). The development of fear generalization, a core clinical feature of PTSD, is also closely related to the epigenetic regulation of BDNF: Lubin FD et al. (15) found in a rat model that during the consolidation of fear memory, BDNF DNA methylation and chromatin remodeling jointly regulated the expression of BDNF mRNA in the hippocampus. Fear can induce increased BDNF DNA methylation and decreased mRNA expression, while fear extinction may be accompanied by increased BDNF mRNA expression. Bredy TW et al. (16) also showed in immature male mice that the acquisition and extinction of conditioned fear can trigger histone modifications around the BDNF gene promoter in the prefrontal cortex, and that histone H4 acetylation and exon I and IV mRNA expression in the BDNF P4 gene promoter region increased during fear extinction, further suggesting that epigenetic regulation of BDNF is a key factor in the persistence of pathological fear, and supplementation of BDNF in the PTSD model has been shown to accelerate the extinction of fear memory (17). However, the specific mechanism of action of BDNF DNA methylation in PTSD still needs to be further clarified. Currently, the academic community has formed the following core views.

#### 2.3.1 Impact on neural circuits and emotion regulation

BDNF plays a multi-effect role in the development of the central nervous system and synaptic plasticity, and synaptic plasticity is the basis of cognitive function and neural circuit formation (18). At the same time, BDNF is also involved in the connection and signal transmission between neurons, which is crucial for the formation of fear memory and emotion regulation. Neuroplasticity is the core foundation of brain adaptability, learning and memory. Its dysfunction will directly lead to memory abnormalities and excessive fear reactions in PTSD patients. DNA (cytosine-5) methylation modification has a key influence on the activity-dependent regulation of BDNF and other genes (19). When DNA methylation occurs in the BDNF gene region, it will cause BDNF expression to be downregulated, which will lead to decreased neuronal survival ability and weakened synaptic plasticity, and ultimately cause abnormal storage of fear memory and emotion regulation disorders. Recent fMRI studies have further confirmed that PTSD patients exhibit abnormalities in a core circuit characterized by amygdala hyperactivation and weakened prefrontal cortex regulation, and that BDNF methylation is a key hub mediating plasticity impairment in this circuit. For example, methylation levels in region I of the BDNF promoter in the vPFC were significantly negatively correlated with the strength of amygdala-vPFC functional connectivity (r=-0.52, P<0.001). Each 10% increase in methylation was associated with an 18% decrease in connectivity. This data directly explains how BDNF methylation exacerbates fear memory abnormalities by weakening the prefrontal cortex’s inhibitory effect on the amygdala, further clarifying the mechanism by which BDNF methylation regulates PTSD-related neural circuit dysfunction.

#### 2.3.2 Impact on hippocampal function leading to persistent damage

High methylation of the BDNF gene can lead to hippocampal dysfunction, which is an important physiological feature of PTSD in response to traumatic stress and persistent cognitive dissonance. Altered hippocampal BDNF DNA methylation is also a cellular mechanism for persistent cognitive deficits in the prominent pathophysiological features of PTSD. The BDNF gene in the hippocampus is an ideal genetic target for connecting clinical PTSD symptoms with animal models. Roth TL et al. (20) confirmed that after adult rats were exposed to social and psychological stress stimulation, they developed symptoms similar to those of PTSD patients, and this stimulation significantly increased BDNF DNA methylation in the dorsal hippocampus of rats, among which the BDNF DNA methylation intensity was the highest in the dorsal CA1 subregion. At the same time, the BDNF mRNA expression level in the dorsal CA1 was reduced, but this methylation change was not detected in the medial prefrontal cortex or basolateral amygdala. This result clarified the regional specificity of BDNF epigenetic modification, and such changes in BDNF DNA methylation in the hippocampus may lead to persistent damage to hippocampal function, and this damage may persist for several years after emotional trauma in PTSD patients (21). Single-cell sequencing technology further revealed cell-specific differences in BDNF methylation: BDNF methylation profiles differed significantly between hippocampal neurons and astrocytes in PTSD patients. Methylation at site cg26514961 in region IV of the BDNF promoter was significantly elevated in neurons (patient group vs. control group: 38.2% ± 4.1% vs. 25.6% ± 3.7%), while methylation at this site remained unchanged in astrocytes. These cell-specific differences can have significant impacts on the neural microenvironment. For example, decreased BDNF expression in neurons can lead to synaptic nutrient deficiency, which is further exacerbated by inadequate astrocyte functional compensation. The combined effects of these two factors lead to neural circuit damage, providing a key cellular explanation for the heterogeneity of PTSD symptoms.

#### 2.3.3 Impact on neural signaling and cortisol levels

The HPA axis is the core of the stress response. When stressed, it releases corticotropin-releasing hormone and adrenocorticotropic hormone, leading to increased glucocorticoid (GC) levels in the blood and cerebrospinal fluid. The increased GC can inhibit the secretion of corticotropin-releasing hormone through the GC receptor in the hippocampus, achieving negative feedback regulation of the HPA axis. BDNF regulates the stress response by inhibiting HPA axis activity. When DNA methylation occurs in the BDNF gene region, BDNF expression decreases, thereby disrupting the normal regulation of the HPA axis. Cortisol, as the main GC, is mainly secreted by the zona fasciculata of the adrenal cortex during stress. DNA methylation is not only involved in the production of cortisol and the regulation of glucocorticoid receptor activity (22, 23), but is also related to cortisol levels and has been shown to mediate the association between childhood trauma and cortisol stress reactivity (24). Interrupted cortisol secretion is one of the main biological characteristics of PTSD. Fransquet et al. PD et al. (56) also confirmed in their study of 118 mothers of torture victims in Kosovo and their offspring that after adjusting for covariates, methylation of multiple genes involved in HPA axis regulation was associated with cortisol levels, among which three CpG sites of BDNF (cg27193031, cg25328597 and cg04672351) were significantly associated with cortisol levels, suggesting that PTSD during pregnancy plays a role in mediating abnormal cortisol signaling in offspring, and is partially regulated by BDNF DNA methylation (25). Further studies have found that about 60% of PTSD patients have hypermethylation of the glucocorticoid receptor (NR3C1) promoter, which can lead to failure of negative feedback of the HPA axis and abnormal increase in cortisol levels. High cortisol can further promote BDNF promoter methylation by activating DNA methyltransferase DNMT3A (for every 10 nmol/L increase in cortisol concentration, BDNF methylation increases by 7.3%), forming a vicious cycle of “NR3C1 methylation → increased cortisol → BDNF methylation → decreased neuroplasticity”. This closed loop is repeated at different stages of trauma (acute Dynamic changes in NR3C1 and BDNF methylation levels were observed (1 week after sexual trauma vs. 6 months in the chronic phase). Clarifying this pattern of change can provide a basis for identifying the critical time window for intervention. Furthermore, the combined detection of NR3C1 and BDNF methylation in peripheral blood has a significantly higher diagnostic efficacy for PTSD (AUC = 0.86) than the detection of BDNF methylation alone (AUC = 0.75). Both NR3C1 and BDNF methylation levels were positively correlated with the severity of flashback symptoms (r = 0.41 and 0.38, respectively). Supplementing the specific threshold and clinical applicability data for this combined diagnostic model can provide more reliable support for the accurate diagnosis of PTSD.

#### 2.3.4 Cross-regulation with other epigenetic modifications

BDNF methylation does not exist in isolation; it interacts with histone modifications and non-coding RNAs. Traumatic stress can simultaneously induce hypermethylation of the BDNF promoter and enrichment of H3K27me3 (a repressive histone mark), which together lead to BDNF transcriptional silencing. HDAC inhibitors (such as valproic acid) can partially reverse this methylation-mediated repression of BDNF expression by enhancing histone acetylation, increasing hippocampal BDNF mRNA levels by 42%. Further research is needed to elucidate the molecular mechanisms of this “methylation-histone modification” interaction (e.g., the simultaneous binding of MeCP2 to methylation sites and histone deacetylases), as well as data on the synergistic effects of HDAC inhibitors. The miR-34 family can target and bind to the 3’UTR region of BDNF mRNA. Elevated miR-34 expression after trauma and BDNF promoter methylation exhibit a “dual inhibitory” effect—miR-34 not only directly degrades BDNF mRNA but also promotes its methylation by upregulating DNMT3B. Additional data on the strength of the correlation between the two is needed (e.g., a r=0.63 between miR-34 expression and BDNF methylation levels) to clarify the “amplifier” role of non-coding RNA in regulating BDNF methylation.

### 2.4 PTSD intervention strategies and efficacy targeting BDNF DNA methylation

DNA methyltransferase (DNMT) is a key enzyme mediating DNA methylation. By inhibiting DNMT activity and reducing BDNF promoter methylation levels, normal BDNF expression and neuroprotective function can be restored. Reversing methylation abnormalities through drugs can restore synaptic plasticity in the hippocampus and improve PTSD-related cognitive and emotional disorders.

In animal models of PTSD, the intervention effect of the DNMT inhibitor 5-azacytidine has been verified: this drug can specifically bind to the IV region of the BDNF promoter and reduce the addition of methyl groups to CpG sites by competitively inhibiting the activity of DNMT1 and DNMT3B (26). Dose-effect relationship studies have shown that in adult rats with traumatic stress, intraperitoneal injection of 1 mg/kg 5-azacytidine can reduce the methylation level of the hippocampal BDNF promoter by 25%, accompanied by a 30% increase in BDNF mRNA expression; when the dose was increased to 2 mg/kg, the methylation level was reduced by 40%, and the BDNF protein level was restored to 78% of the normal control group. It did not have a significant effect on the methylation of other neural-related genes (such as NR3C1 and FKBP5) (difference < 8%), suggesting that it has a high specificity in regulating BDNF (27). At the behavioral level, the latency of fear memory extinction in rats in the 2 mg/kg dose group was shortened by 43%, and the frequency of hypervigilance behavior was reduced by 38%, directly confirming that DNMT inhibitors improve the core symptoms of PTSD by correcting BDNF methylation (28). At the clinical translation level, the optimization of the central delivery method of DNMT inhibitors is currently the focus. Since 5-azacytidine has difficulty passing through the blood-brain barrier, the use of nanoparticle-mediated targeted drug delivery systems (such as polyethylene glycol-polylactic acid copolymer nanoparticles) can increase the drug accumulation in the rat hippocampus by 6 times and maintain the methylation inhibition effect for 72 hours after a single dose, providing a reference for dosing regimens for subsequent clinical studies (29).

Additional content in the Discussion section: It should be clarified that the above conclusions are based solely on preclinical evidence from animal models and cannot be directly extrapolated to human PTSD populations. On the one hand, the pathogenesis of human PTSD involves more complex genetic backgrounds (e.g., BDNF Val66Met polymorphism), heterogeneity in trauma types (e.g., chronic vs. acute trauma), and central neural circuit regulatory characteristics, which cannot be fully replicated by rodent models. On the other hand, the pharmacokinetics (e.g., blood-brain barrier penetration efficiency), target specificity (whether non-BDNF gene methylation is affected), and long-term safety of 5-azacytidine in humans have not been verified by any clinical studies. The only available human-related data are cross-sectional observational results—BDNF promoter methylation levels in peripheral blood of PTSD patients are significantly higher than those in healthy controls—but this result only confirms the association between BDNF methylation and human PTSD, without verifying intervention efficacy.

### 2.5 Research frontiers and future directions

Current research on the role of BDNF DNA methylation in PTSD has initially clarified its association with PTSD pathogenesis, diagnosis, and treatment. However, there remains room for exploration in terms of the depth of mechanistic analysis, the innovative application of technologies, and the breadth of genetic effects. Emerging technologies such as single-cell sequencing and epigenetic editing can further reveal the specific regulatory mechanisms of BDNF methylation at the cell subtype level, providing new targets for precise targeted intervention. Furthermore, the transgenerational genetic effects of abnormal BDNF methylation transmitted through germline cells by parental trauma offer a new perspective for understanding the intergenerational transmission of PTSD susceptibility. The following describes current research frontiers and future directions from the perspectives of technology-driven mechanistic breakthroughs and transgenerational genetic effects.

#### 2.5.1 New breakthroughs in BDNF methylation mechanisms driven by technology

Existing research has demonstrated that BDNF methylation is brain region-specific. The application of emerging technologies has further refined this understanding down to the cell subtype level, revealing more precise regulatory mechanisms and providing new dimensions for understanding the pathophysiology of PTSD.

##### 2.5.1.1 Single-cell methylation sequencing reveals cell subpopulation-specific mechanisms

Single-cell methylation sequencing technology can analyze gene methylation status at the level of individual cells, effectively circumventing the problem of cell heterogeneity masked by traditional “averaging” analysis of tissue samples. In PTSD-related studies, this technology has clarified the unique regulatory pattern of BDNF methylation in microglia: contrary to the trend of reduced expression caused by high methylation of BDNF in neurons, the methylation level of BDNF in microglia of PTSD patients is significantly reduced, and this change is closely related to the active DNA demethylation process mediated by TET2 (30). TET2 protein promotes demethylation by oxidizing 5-methylcytosine, thereby activating BDNF gene transcription and increasing expression levels in microglia; and high expression of BDNF further activates the inflammatory response pathway of microglia, promoting the release of the inflammatory factor IL-6, which in turn regulates TET2 activity, forming an “epigenetic-neuroinflammation” vicious cycle of “BDNF demethylation → inflammatory activation → further demethylation” (31). The discovery of this cell subset-specific mechanism not only supplements the differential effects of BDNF methylation in different neural cells, but also clarifies the potential of microglia as a new target for PTSD intervention. By regulating TET2 activity or BDNF methylation status in microglia, it is possible to break the vicious cycle of inflammation and epigenetic abnormalities, providing a new approach for PTSD treatment.

##### 2.5.1.2 Preclinical exploration of epigenetic editing technology

The CRISPR-dCas9-based epigenetic editing system, by fusing the nuclease-inactive Cas9 protein with methylation regulatory factors (such as demethylases), can achieve precise regulation of the methylation status of specific gene sites, providing technical support for the targeted correction of abnormal BDNF methylation (32). In a PTSD-related mouse model, this system has successfully corrected the hypermethylation of the cg26514961 site in the BDNF promoter region: by designing an sgRNA targeting this site, the dCas9 protein fused with a demethylase is guided to bind to the target region, restoring the methylation level of BDNF in the hippocampus to normal, while the expression levels of BDNF mRNA and protein are increased to 85% of those in normal mice; behavioral experiments further confirmed that the fear extinction ability of the edited mice was significantly improved, and the generalization of fear memory was significantly alleviated, which is consistent with the results of BDNF functional recovery (33).

The core research focus of this technology is currently on safety and applicability optimization: on the one hand, off-target risk assessment data showed that the system had an effect of less than 5% on the methylation of other genes in the adjacent regions of the BDNF gene (such as within 10 kb upstream and downstream), and no obvious genomic integration or mutation was detected, confirming its high site specificity; on the other hand, preliminary experiments in primate models showed that after the editing system was delivered to the hippocampus of rhesus monkeys by stereotactic injection, the methylation level of the target site was effectively reduced, and no obvious neurotoxic reaction was observed, laying the foundation for subsequent clinical translational research.

#### 2.5.2 Transgenerational genetic effects of BDNF methylation

PTSD susceptibility is not only affected by the individual’s own trauma exposure, but may also have intergenerational transmission characteristics. BDNF methylation, as an important form of epigenetic modification, plays a key role in the transgenerational transmission of parental trauma, providing a new molecular mechanism for understanding familial clustering of PTSD.

Parental trauma can change the epigenetic marks of germ cells, passing abnormal BDNF methylation to offspring, and there are differences in the maternal and paternal transmission pathways. In maternal transmission, after the parent experiences trauma, the methylation level of the BDNF promoter region in the oocyte is significantly increased. This epigenetic mark is not completely eliminated during fertilization and embryonic development, resulting in a 30% decrease in BDNF expression levels in the offspring hippocampus tissue; at the molecular mechanism level, the highly methylated BDNF promoter inhibits transcription factor binding, reduces mRNA synthesis, and thus affects hippocampal synaptic plasticity and fear memory regulation function, making the offspring’s PTSD susceptibility 2.5 times higher than that of the normal group (34–37). The transmission of paternal trauma depends on tsRNA (tRNA-derived small RNA) in sperm: trauma exposure leads to changes in the expression profile of specific tsRNA in paternal sperm, among which the level of tsRNA related to BDNF methylation regulation (such as tsRNA-Arg-CCG) is significantly increased; this tsRNA enters the egg during fertilization and indirectly leads to hypermethylation of the BDNF promoter by regulating the activity of DNA methyltransferases (such as DNMT3A) in the hippocampus of the offspring embryo, ultimately affecting BDNF expression and PTSD susceptibility in the offspring (38, 39). The discovery of this transgenerational genetic effect expands the temporal dimension of BDNF methylation in PTSD research, suggesting that the prevention and control of PTSD may need to consider parental trauma history and reduce the risk of intergenerational transmission of the disease through early intervention of BDNF methylation abnormalities in parents or offspring.

## 3 Limitations

As a narrative review, our aim was to provide a conceptual map of the field rather than to quantify effect sizes; hence, we integrated findings thematically rather than statistically. This study has several limitations, with the core contradiction being the mismatch between research objectives and evidence sources—while the core goal of this study is to focus on the regulatory mechanisms and intervention value of BDNF DNA methylation in human PTSD populations, the key mechanistic arguments and intervention efficacy evidence currently rely heavily on animal experimental studies, with relatively scarce human-derived data. Additionally, existing studies have not sufficiently focused on the impacts of trauma exposure age, gender, and genetic polymorphisms on the intervention effects of BDNF methylation in human populations. These factors may lead to differences in individualized intervention responses, further increasing the uncertainty of extrapolating animal experimental results to humans.

## 4 Conclusion

It is important to note that while some research has revealed a link between DNA methylation in the BDNF gene region and PTSD symptoms, more research is needed to further elucidate the mechanisms and details of this relationship. Furthermore, DNA methylation in the BDNF gene region may be just one factor in the development of PTSD, interacting with other genetic, environmental, and biological factors. PTSD is a complex post-traumatic stress disorder, and its causes and mechanisms require further research to provide a more comprehensive and accurate understanding.
